# Supplementary material for: SIRT4 positively regulates autophagy via ULK1, but independently of HDAC6 and OPA1
Source: FEBS Open Bio. 2025 Nov 20;16(5):870–84. doi: 10.1002/2211-5463.70164 (PMC13145358; doi:10.1002/2211-5463.70164)
Supplement: Supplementary file 1 — Fig. S1. CoCl2 treatment induces a pseudo‐hypoxic response in wild‐type HEK293 cells. Fig. S2. Generation and characterization of HEK293 cell lines stably expressing C‐terminal eGFP fusion or myc‐Flag tagged versions of SIRT4 or SIRT4(H161Y). Fig. S3. Inhibitory impact of SIRT4(H161Y) on autophagic flux upon CoCl2‐induced pseudohypoxia. Fig. S4. SIRT4(H161Y) inhibits autophagic flux upon CCCP/oligomycin‐mediated mitochondrial stress or Rapamycin treatment. Fig. S5. Immunoblot analysis of acetylated α‐tubulin (K40) protein levels in CoCl2‐treated HEK293 cell lines expressing eGFP‐fused (A) or myc‐Flag‐tagged (B) SIRT4 or SIRT4(H161Y) in the presence or absence of the HDAC6 inhibitor tubacin. Fig. S6. Impact of tubacin or MYLS22 treatment on the inhibited autophagic flux of CCCP/oligomycin‐treated HEK293‐SIRT4(H161Y) cells. Fig. S7. Impact of tubacin or MYLS22 treatment on the inhibited autophagic flux of rapamycin‐treated HEK293‐SIRT4(H161Y) cells. Fig. S8. ShRNA‐mediated downregulation of SIRT4 expression does not translate into a decreased LC3B‐II response upon CoCl2 treatment. Figs S9–S21. Representative data histograms are depicted for all flow cytometry‐based experiments to measure autophagic flux (GFP‐LC3‐RFP‐LC3ΔG), mitophagy (mt‐mKEIMA), and mitochondrial content (MitoMark). [file FEB4-16-870-s002.pdf]

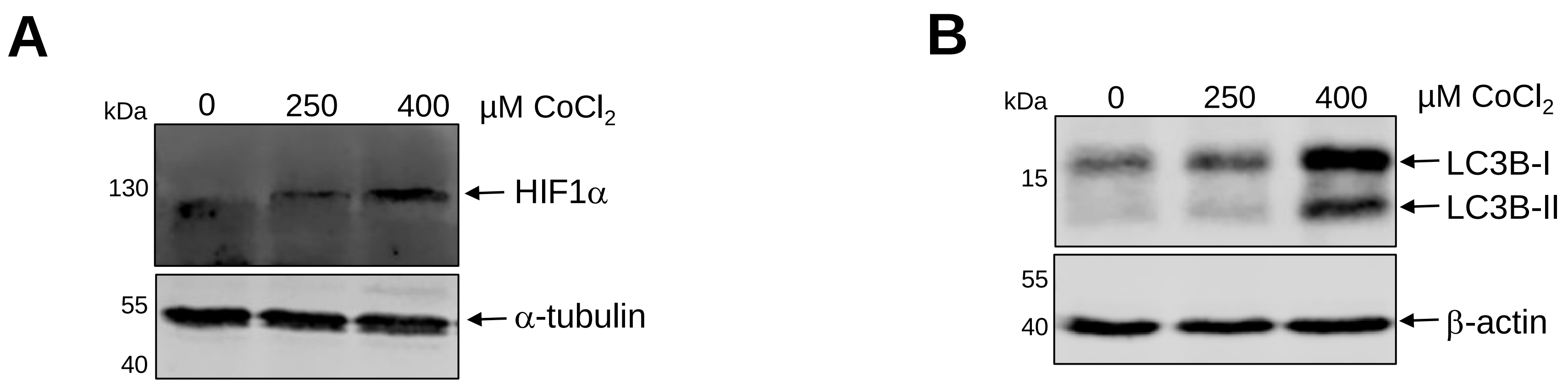

Fig. S1. $CoCl_2$ treatment induces a pseudo-hypoxic response in wild-type HEK293 cells. HEK293 cells were subjected to treatment with 250 μM and 400 μM $CoCl_2$ for 24 h followed by immunoblot analysis of HIF1α (**A**) and LC3B-II (**B**) protein levels.

Figure S1

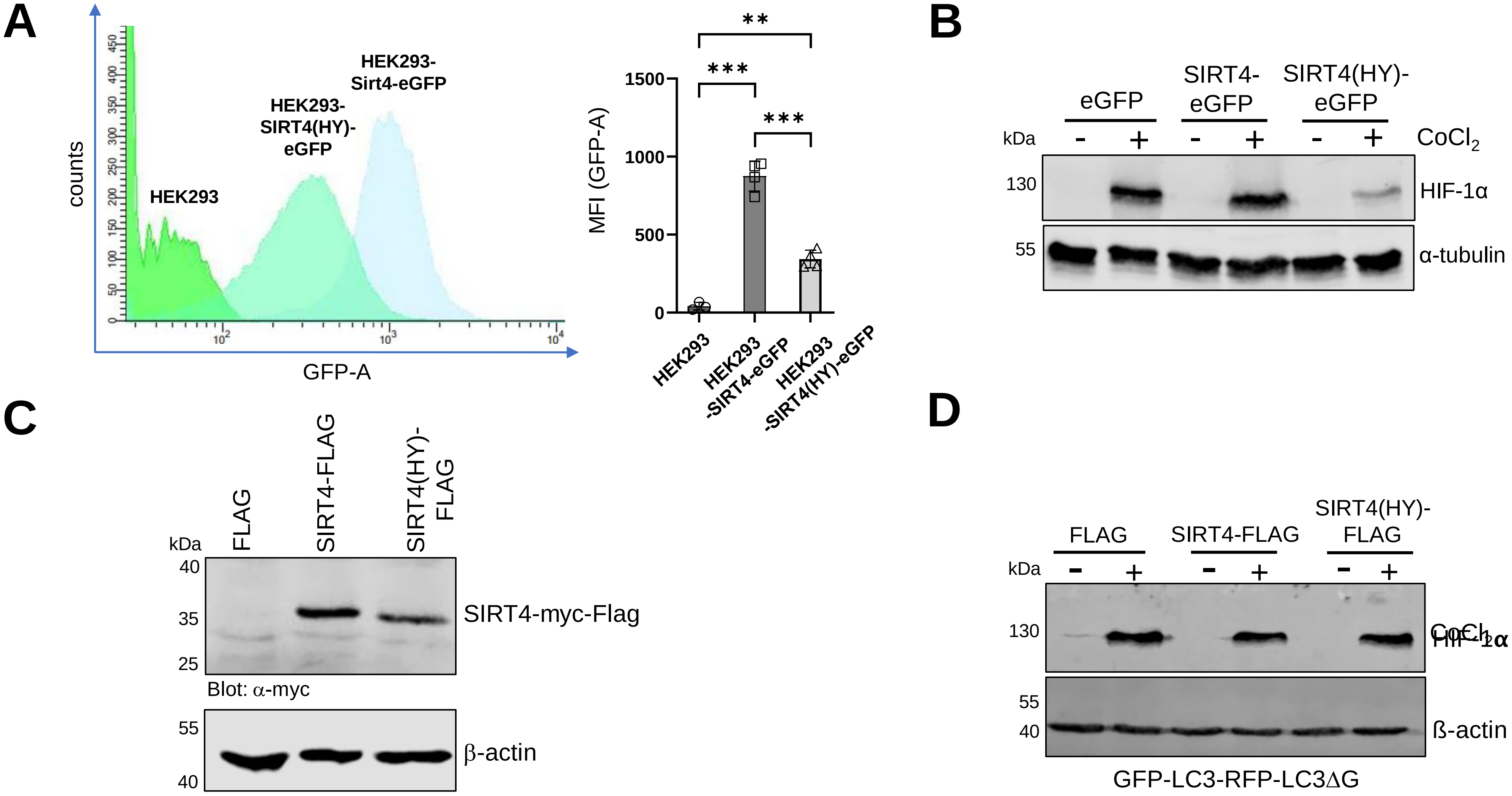

Fig. S2. Generation and characterization of HEK293 cell lines stably expressing C-terminal eGFP fusion or myc-Flag tagged versions of SIRT4 or SIRT4(H161Y). (**A**) Expression levels of SIRT4-eGFP and SIRT4(H161Y)-eGFP were measured by flow cytometry (BD FACS Canto II) and results were analysed using the FlowJo v10 software and calculated as MFI (Median Fluorescence Intensity). (**B**) HEK293 cell lines stably expressing SIRT4-eGFP or SIRT4(H161Y)-eGFP were subjected to treatment with 400 μM $CoCl_2$ for 24 h followed by immunoblot analysis of HIF1α protein levels. (**C**) Analysis of SIRT4-myc-Flag and SIRT4(H161Y)-myc-Flag expression levels in HEK293 cell lines by immunoblotting. (**D**) HEK293 cell lines stably expressing SIRT4-myc-Flag or SIRT4(H161Y)-myc-Flag together with the autophagic flux probe LC3-eGFP were subjected to treatment with 400 μM $CoCl_2$ for 24 h followed by immunoblot analysis of HIF1α protein levels.

Figure S2

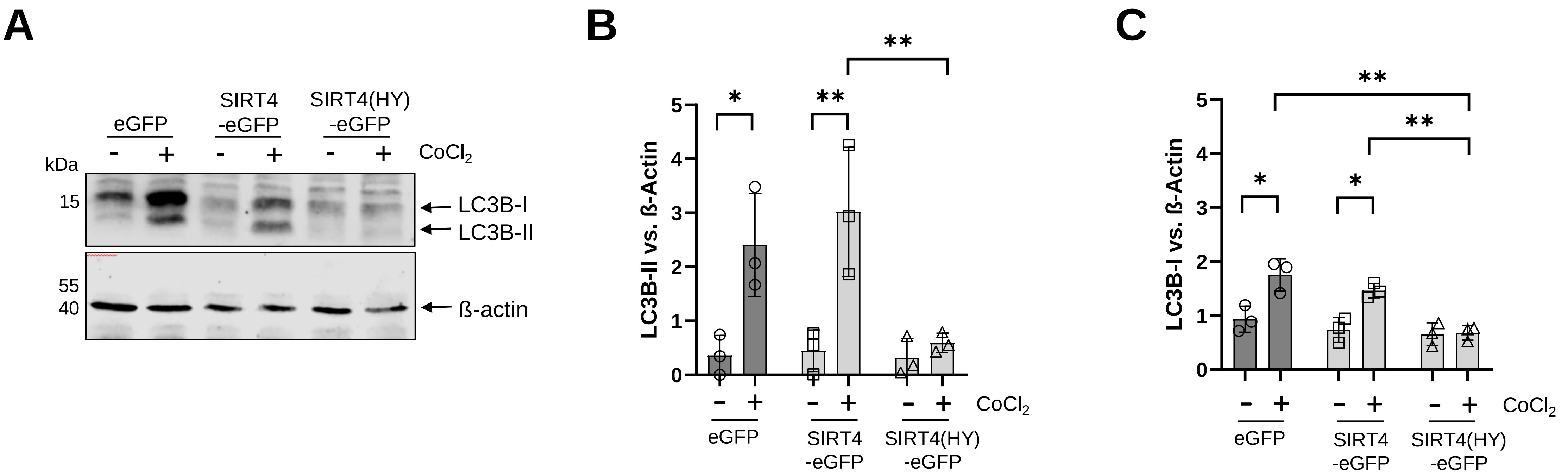

Fig. S3. Inhibitory impact of SIRT4(H161Y) on autophagic flux upon $CoCl_2$-induced pseudohypoxia. (**A**) HEK293 cells stably expressing eGFP, SIRT4-eGFP, or SIRT4(H161Y)-eGFP were subjected to $CoCl_2$ treatment for 36 h followed by immunoblot analysis of LC3B-I/II levels. (**B, C**) Relative quantification of immunoblot signals of LC3B-II and LC3B-I was performed using ImageJ-based densitometric evaluation and β-actin levels as loading control (n=3). To determine statistical significance, Two-Way ANOVA tests were employed (mean ± S.D.; *$p < 0.05$; **$p < 0.01$).

Figure S3

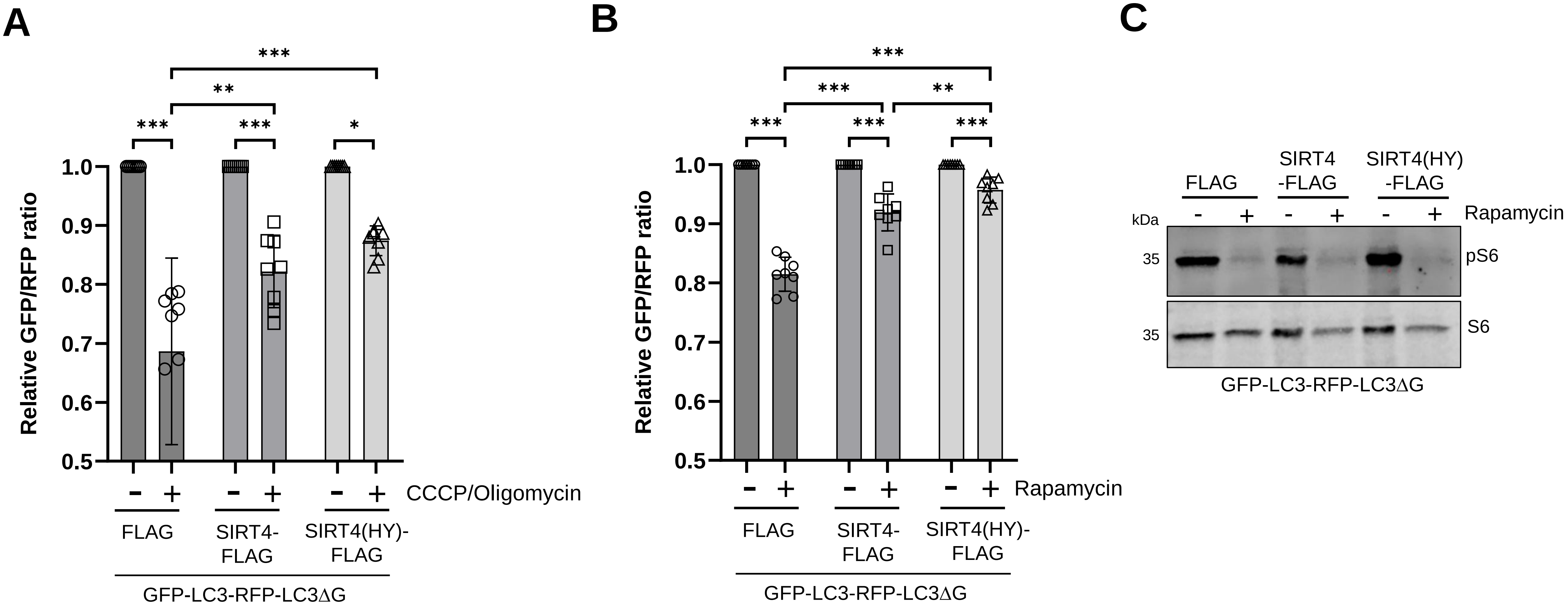

Fig. S4. SIRT4(H161Y) inhibits autophagic flux upon CCCP/oligomycin-mediated mitochondrial stress or Rapamycin treatment. HEK293 cells stably expressing myc-Flag, SIRT4-myc-Flag, or SIRT4(H161Y)-myc-Flag were subjected to CCCP/oligomycin (**A**) or rapamycin (**B**) treatment followed by flow cytometry-based analysis of autophagic flux using the GFP-LC3-RFP-LC3ΔG probe (n=7-8). To determine statistical significance, Two-Way ANOVA tests were employed (mean ± S.D.; $^*p < 0.05$; $^{**}p < 0.01$; $^{***}p < 0.001$). (**C**) HEK293 cell lines stably expressing myc-Flag, SIRT4-myc-Flag or SIRT4(H161Y)-myc-Flag were subjected to treatment with 1.25 μM rapamycin for 24 h followed by immunoblot analysis of pS6 (Ser240/244) and total ribosomal S6 protein levels.

Figure S4

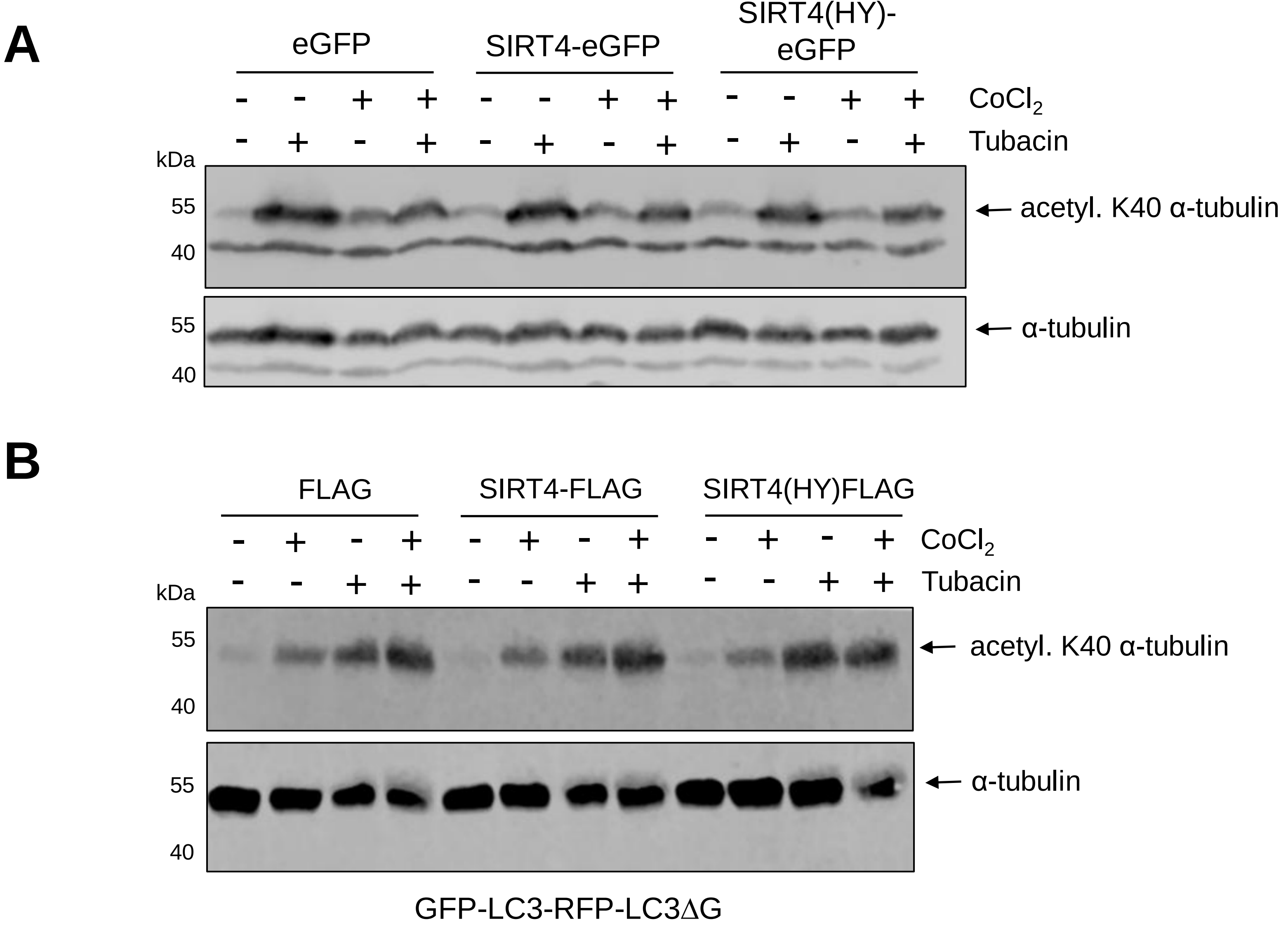

Fig. S5. Immunoblot analysis of acetylated α-Tubulin (K40) protein levels in $CoCl_2$-treated HEK293 cell lines expressing eGFP-fused (**A**) or myc-Flag-tagged (**B**) SIRT4 or SIRT4(H161Y) in the presence or absence of the HDAC6 inhibitor tubacin. Probing against α-tubulin was used as loading control.

Figure S5

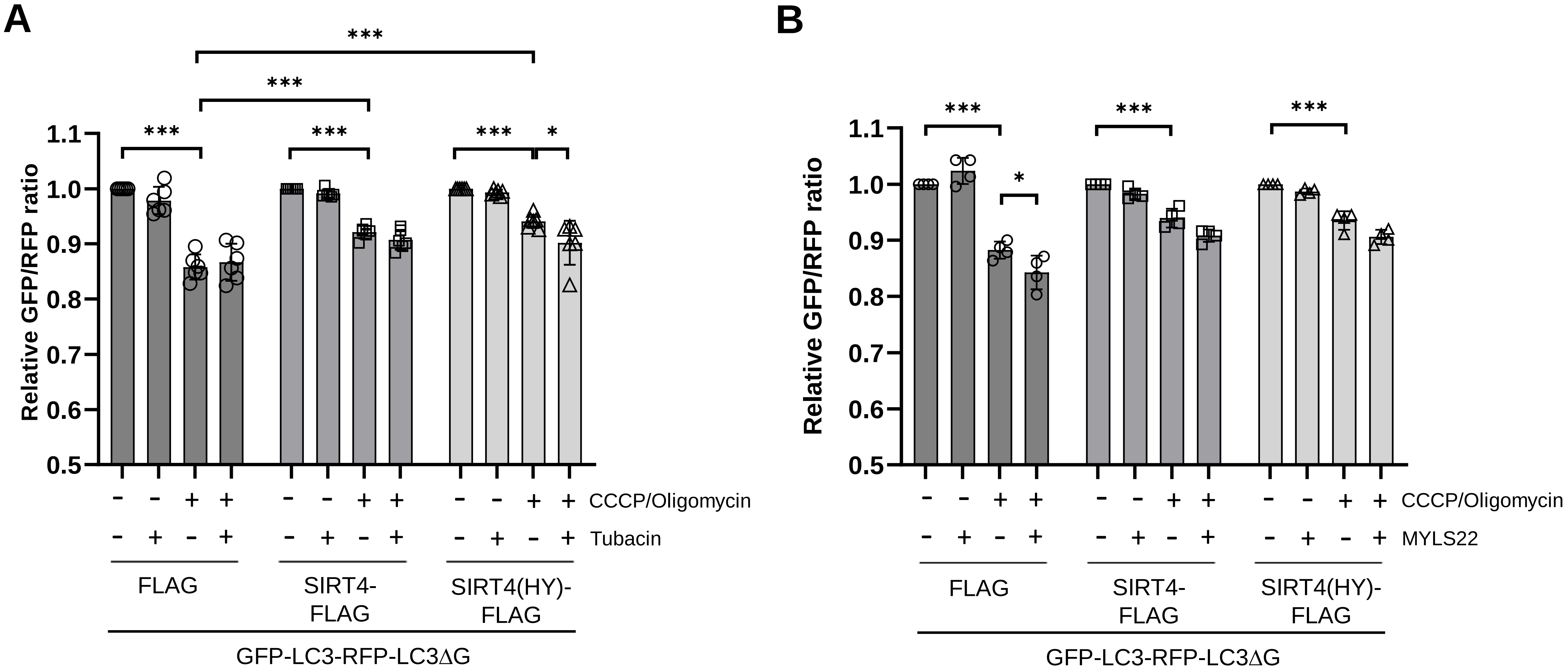

Fig. S6. Impact of tubacin or MYLS22 treatment on the inhibited autophagic flux of CCCP/oligomycin treated HEK293- SIRT4(H161Y) cells. HEK293 cells stably expressing myc-Flag, SIRT4-myc-Flag, or SIRT4(H161Y)-myc-Flag were subjected to CCCP/oligomycin treatment in the presence of tubacin (HDAC6 inhibitor; n=6) (**A**) or MYLS22 (OPA1 inhibitor; n=4) (**B**) followed by flow cytometry-based analysis of autophagic flux using the GFP-LC3-RFP-LC3ΔG probe. To determine statistical significance, Two-Way ANOVA tests were employed (mean ± S.D.; *$p < 0.05$; *** $p < 0.001$).

Figure S6

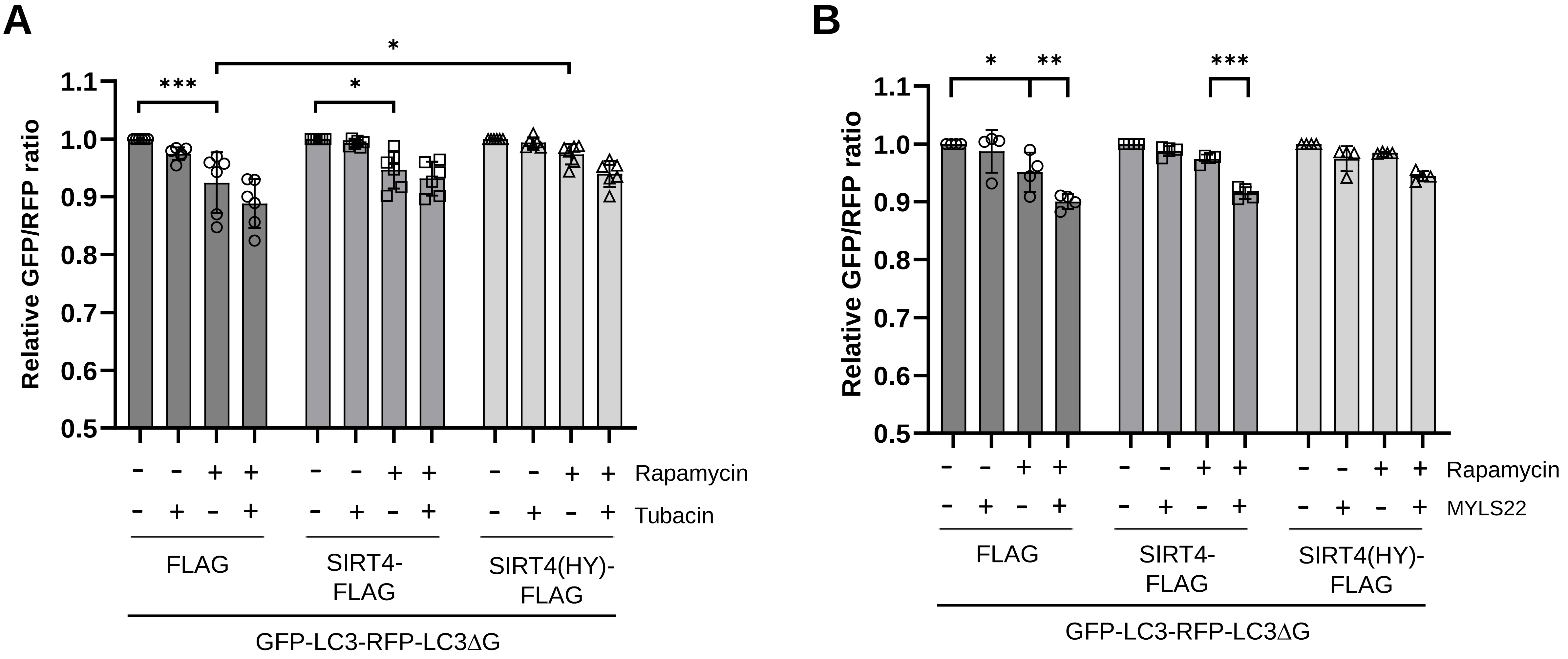

Fig. S7. Impact of tubacin or MYLS22 treatment on the inhibited autophagic flux of rapamycin treated HEK293-SIRT4(H161Y) cells. HEK293 cells stably expressing myc-Flag, SIRT4-myc-Flag, or SIRT4(H161Y)-myc-Flag were subjected to rapamycin treatment in the presence of tubacin (HDAC6 inhibitor; n=6) (**A**) or MYLS22 (OPA1 inhibitor; n=4) (**B**) followed by flow cytometry-based analysis of autophagic flux using the GFP-LC3-RFP-LC3ΔG probe. To determine statistical significance, Two-Way ANOVA tests were employed (mean ± S.D.; *$p < 0.05$; **$p < 0.01$; ***$p < 0.001$).

Figure S7

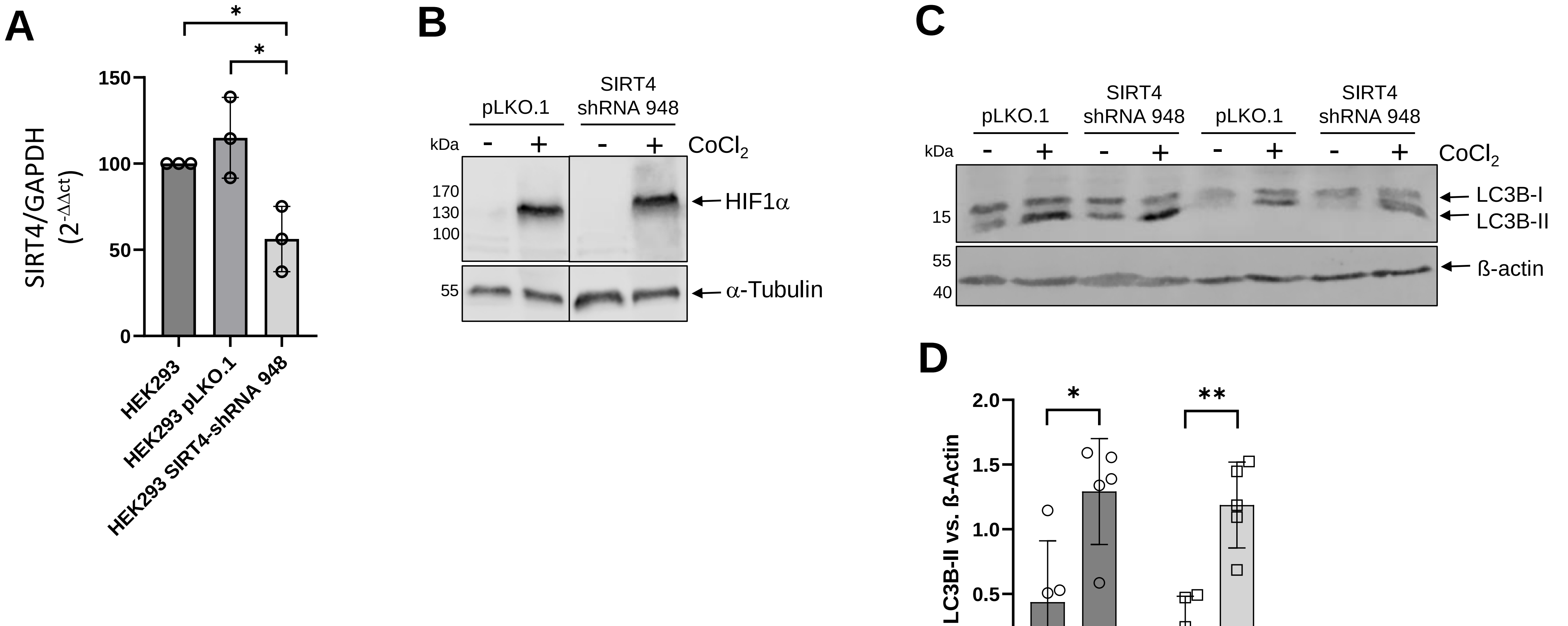

Fig. S8. ShRNA mediated downregulation of SIRT4 expression does not translate into a decreased LC3B-II response upon $CoCl_2$ treatment. (**A**) Real-time PCR analysis of SIRT4 mRNA levels upon shRNA-mediated downregulation of SIRT4 expression. GAPDH was employed as reference mRNA (n=3). To determine statistical significance, One-Way ANOVA tests were employed (mean ± S.D.). (**B**) Normal pseudo-hypoxic response in HEK293 cells with downregulated SIRT4 expression. The indicated HEK293 cell lines were treated with 400 µM $CoCl_2$ for 24 h followed by immunoblot analysis of HIF1α. (**C**) $CoCl_2$-triggered increase of LC3B-II protein levels in HEK293 cells with normal *vs.* downregulated SIRT4 expression. Two independent experiments are depicted. (**D**) Relative quantification of immunoblot signals of LC3B-II was performed using ImageJ-based densitometric evaluation and β-actin levels as loading control (n=5). To determine statistical significance, Two-Way ANOVA tests were employed (mean ± S.D.; *$p < 0.05$; **$p < 0.01$).

Figure S8

ad Figure 1D

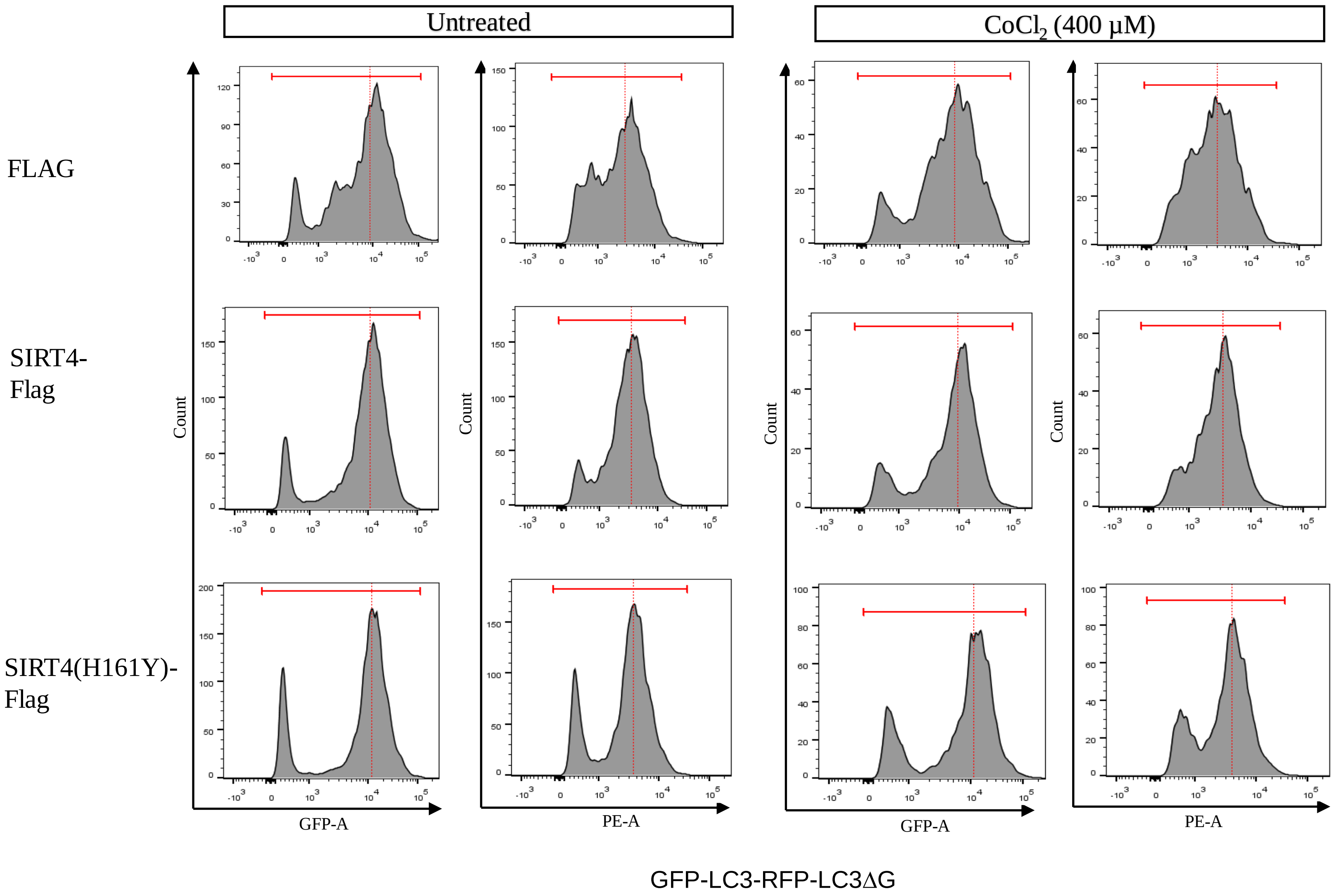

Fig. S9 to S21: Representative data histograms are depicted for all flow cytometry-based experiments to measure autophagic flux (GFP-LC3-RFP-LC3ΔG), mitophagy (mt-mKEIMA), and mitochondrial content (MitoMark). Solid red lines indicate the fluorescence gates chosen for analysis. Red dotted lines represent the median values of emission.

Figure S9

ad Figure 1E

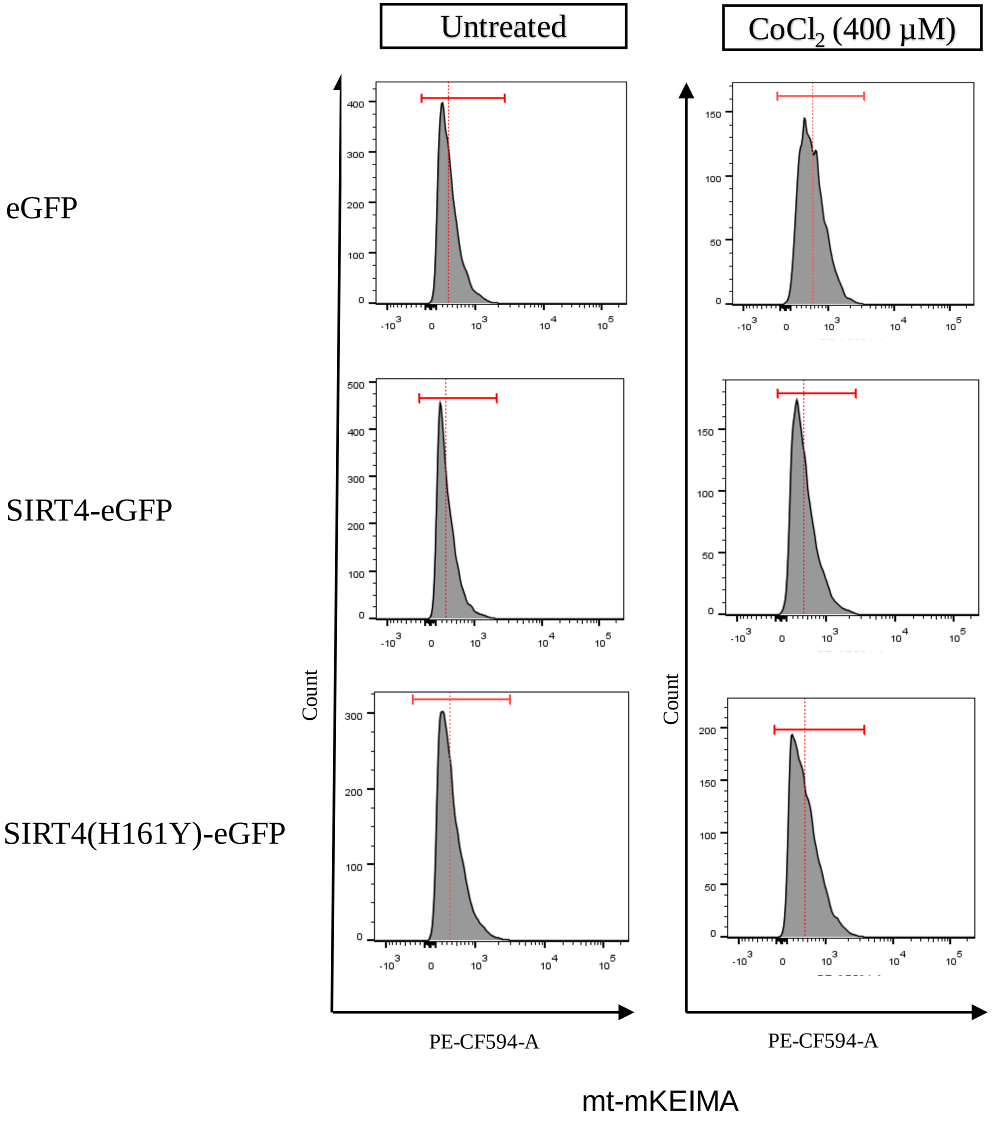

Fig. S9 to S21: Representative data histograms are depicted for all flow cytometry-based experiments to measure autophagic flux (GFP-LC3-RFP-LC3ΔG), mitophagy (mt-mKEIMA), and mitochondrial content (MitoMark). Solid red lines indicate the fluorescence gates chosen for analysis. Red dotted lines represent the median values of emission.

Figure S10

ad Figure 1F

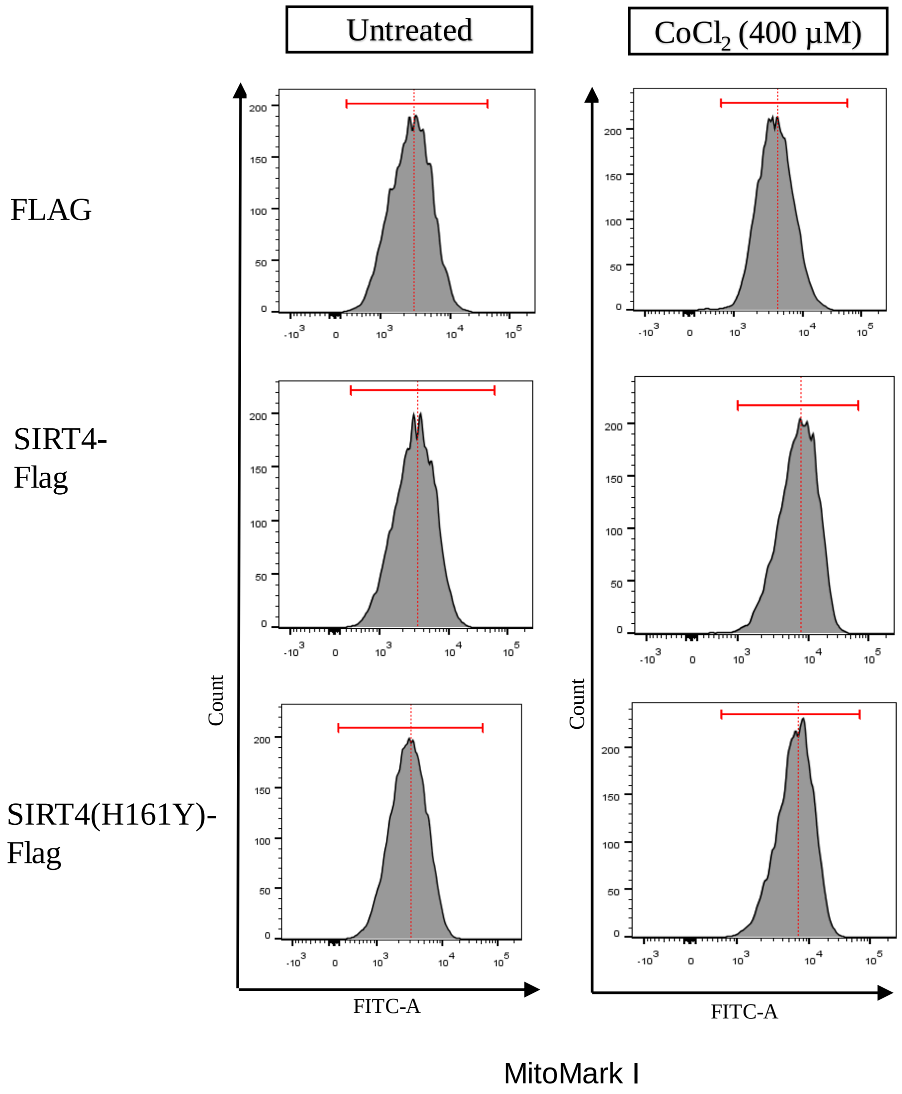

Fig. S9 to S21: Representative data histograms are depicted for all flow cytometry-based experiments to measure autophagic flux (GFP-LC3-RFP-LC3ΔG), mitophagy (mt-mKEIMA), and mitochondrial content (MitoMark). Solid red lines indicate the fluorescence gates chosen for analysis. Red dotted lines represent the median values of emission.

Figure S11

ad Figure 3C

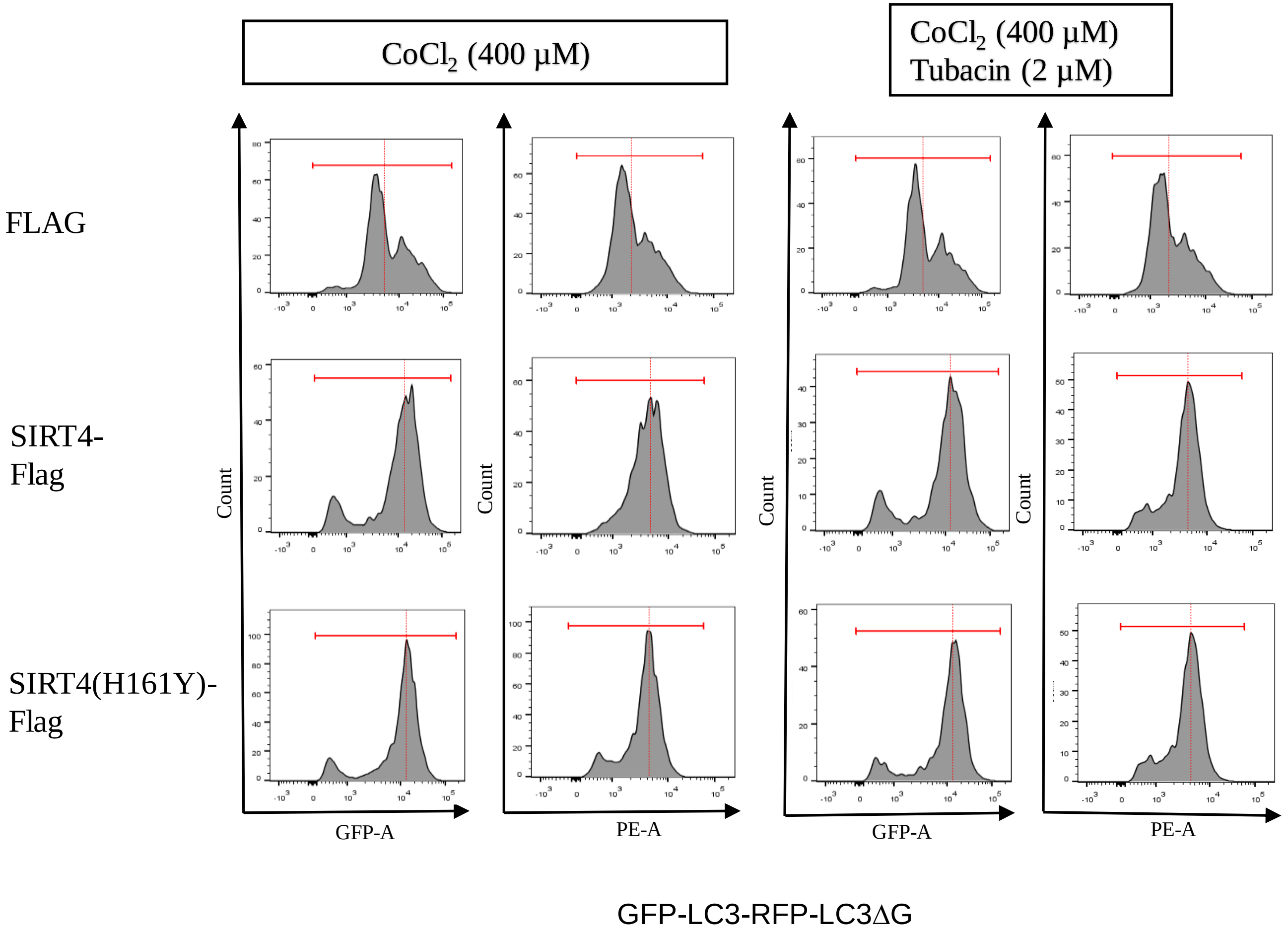

Fig. S9 to S21: Representative data histograms are depicted for all flow cytometry-based experiments to measure autophagic flux (GFP-LC3-RFP-LC3ΔG), mitophagy (mt-mKEIMA), and mitochondrial content (MitoMark). Solid red lines indicate the fluorescence gates chosen for analysis. Red dotted lines represent the median values of emission.

Figure S12

ad Figure 3D

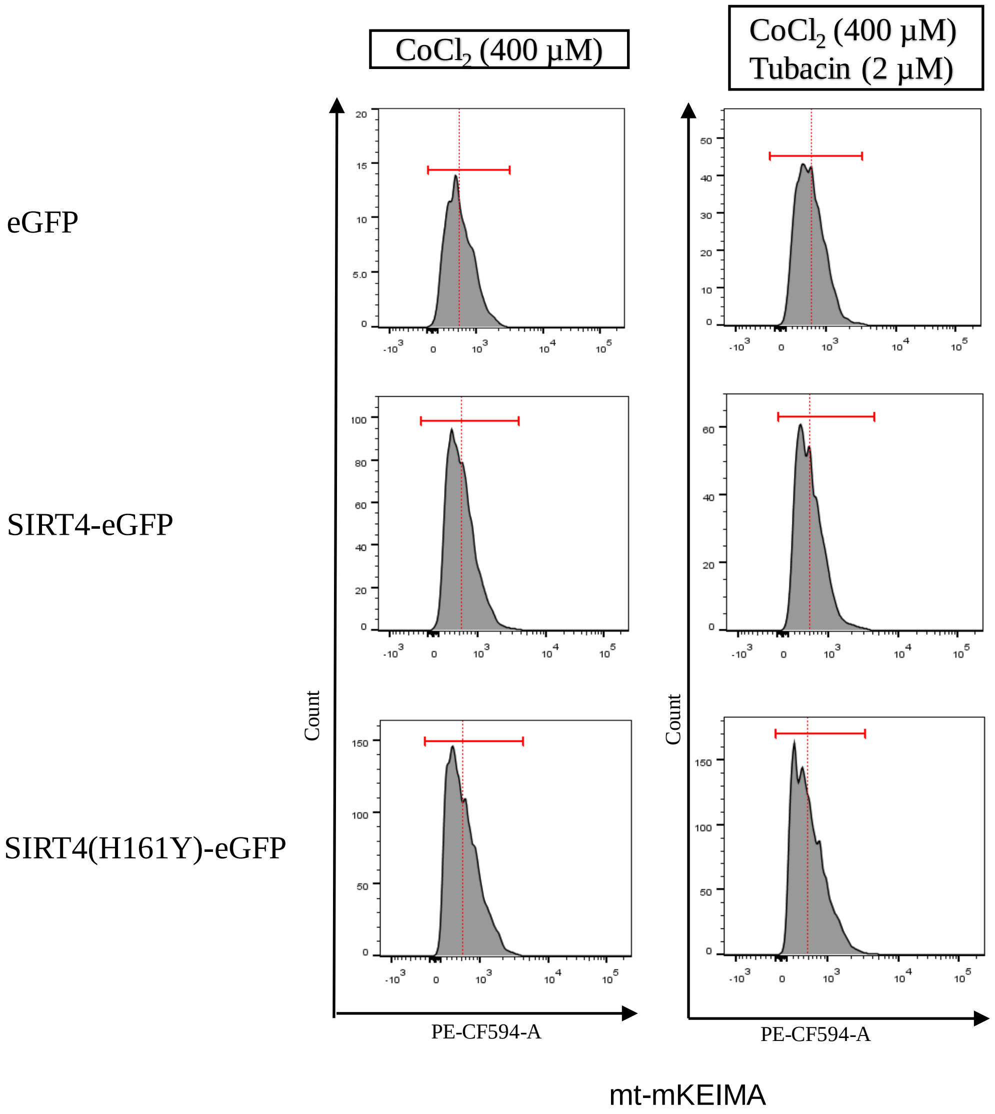

Fig. S9 to S21: Representative data histograms are depicted for all flow cytometry-based experiments to measure autophagic flux (GFP-LC3-RFP-LC3ΔG), mitophagy (mt-mKEIMA), and mitochondrial content (MitoMark). Solid red lines indicate the fluorescence gates chosen for analysis. Red dotted lines represent the median values of emission.

Figure S13

ad Figure 5C

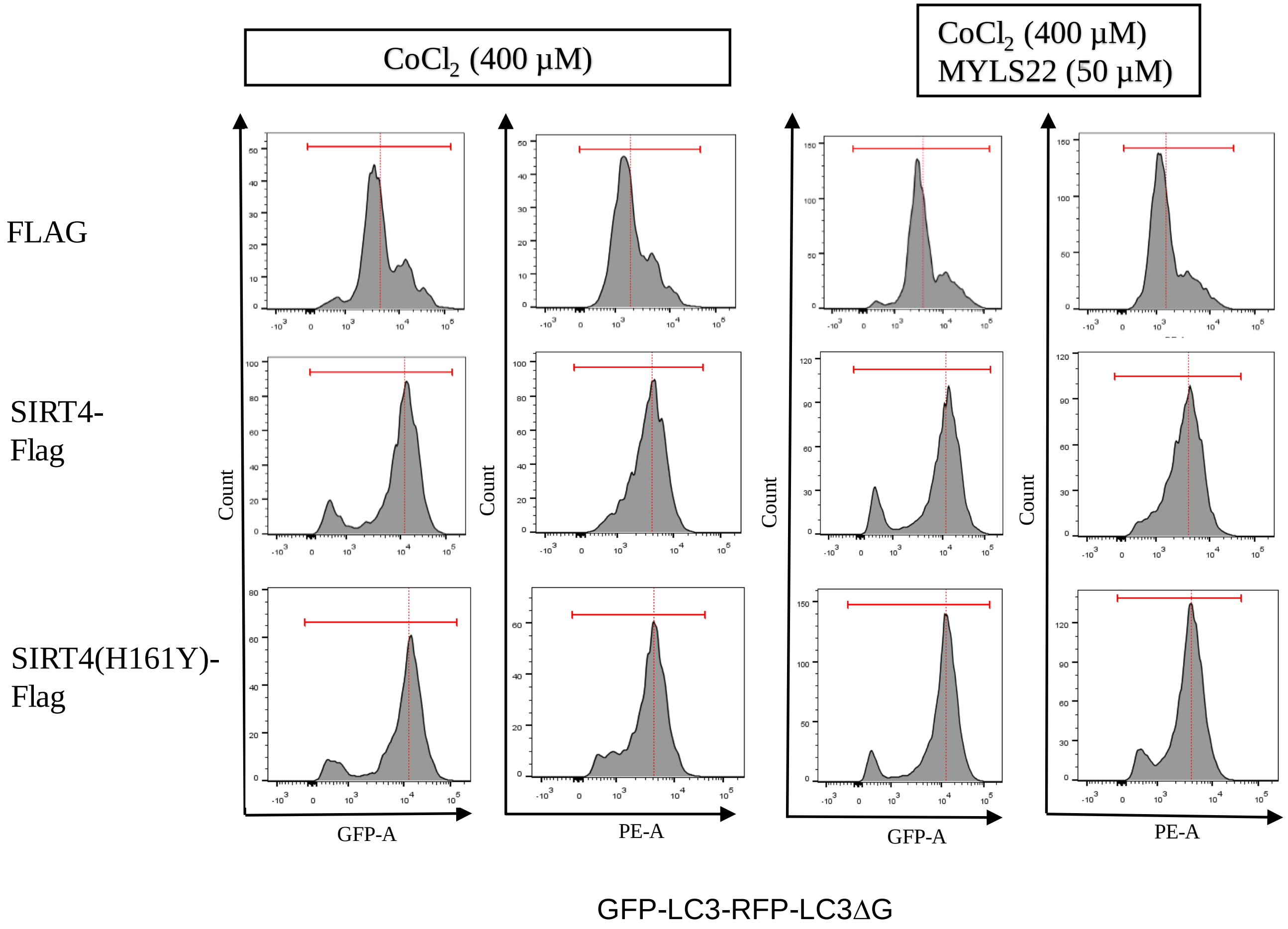

Fig. S9 to S21: Representative data histograms are depicted for all flow cytometry-based experiments to measure autophagic flux (GFP-LC3-RFP-LC3ΔG), mitophagy (mt-mKEIMA), and mitochondrial content (MitoMark). Solid red lines indicate the fluorescence gates chosen for analysis. Red dotted lines represent the median values of emission.

ad Figure 5D

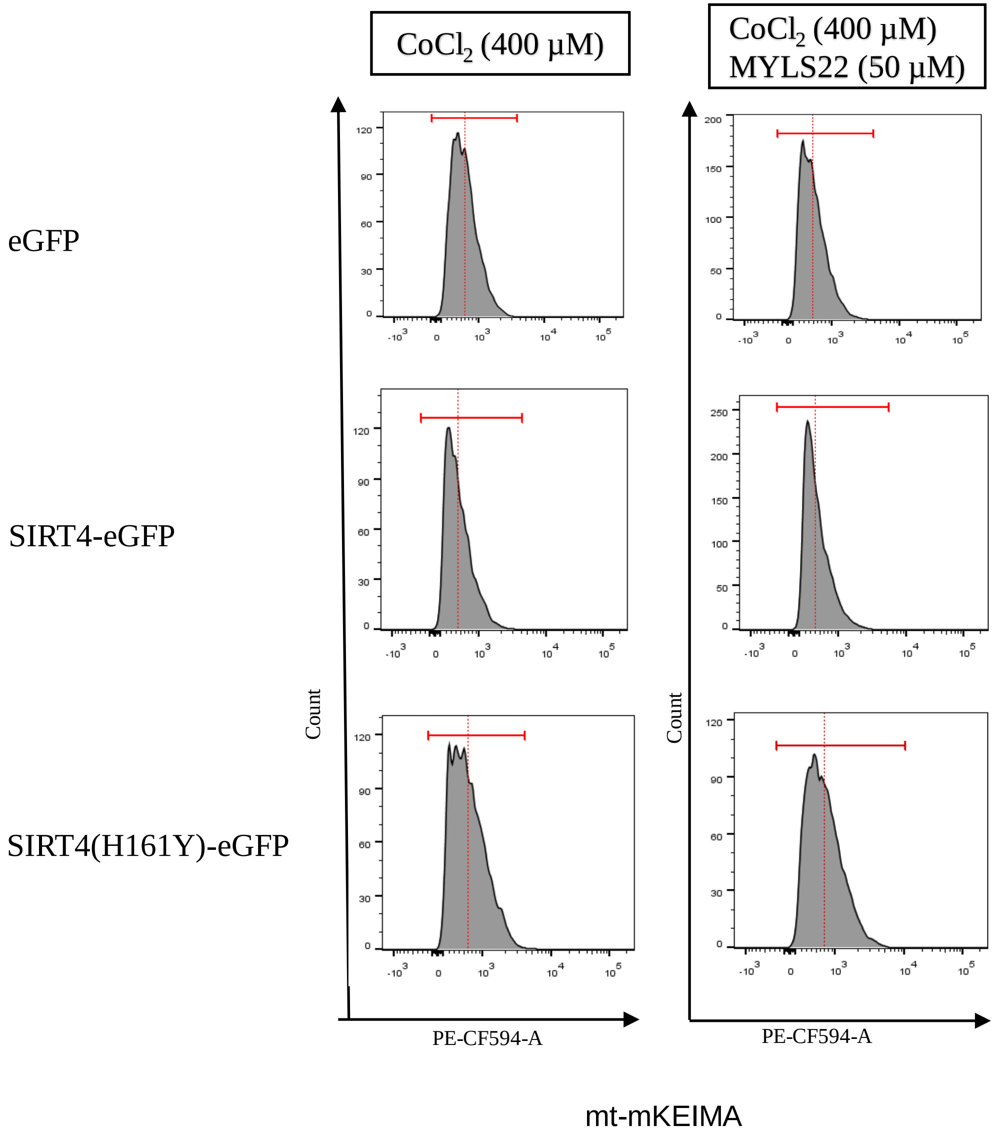

Fig. S9 to S21: Representative data histograms are depicted for all flow cytometry-based experiments to measure autophagic flux (GFP-LC3-RFP-LC3ΔG), mitophagy (mt-mKEIMA), and mitochondrial content (MitoMark). Solid red lines indicate the fluorescence gates chosen for analysis. Red dotted lines represent the median values of emission.

Figure S15

ad Figure S4A

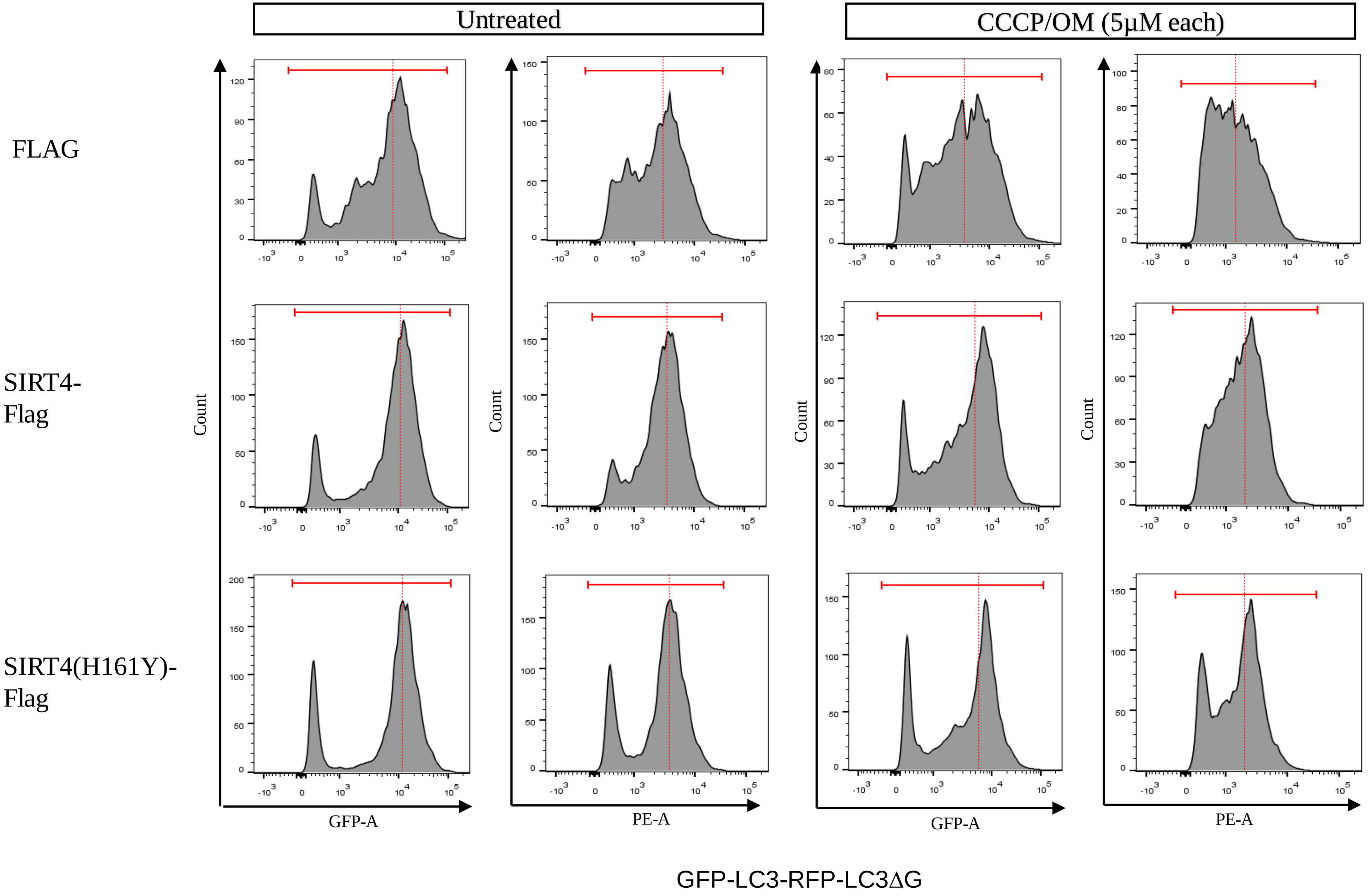

Fig. S9 to S21: Representative data histograms are depicted for all flow cytometry-based experiments to measure autophagic flux (GFP-LC3-RFP-LC3ΔG), mitophagy (mt-mKEIMA), and mitochondrial content (MitoMark). Solid red lines indicate the fluorescence gates chosen for analysis. Red dotted lines represent the median values of emission.

Figure S16

ad Figure S4B

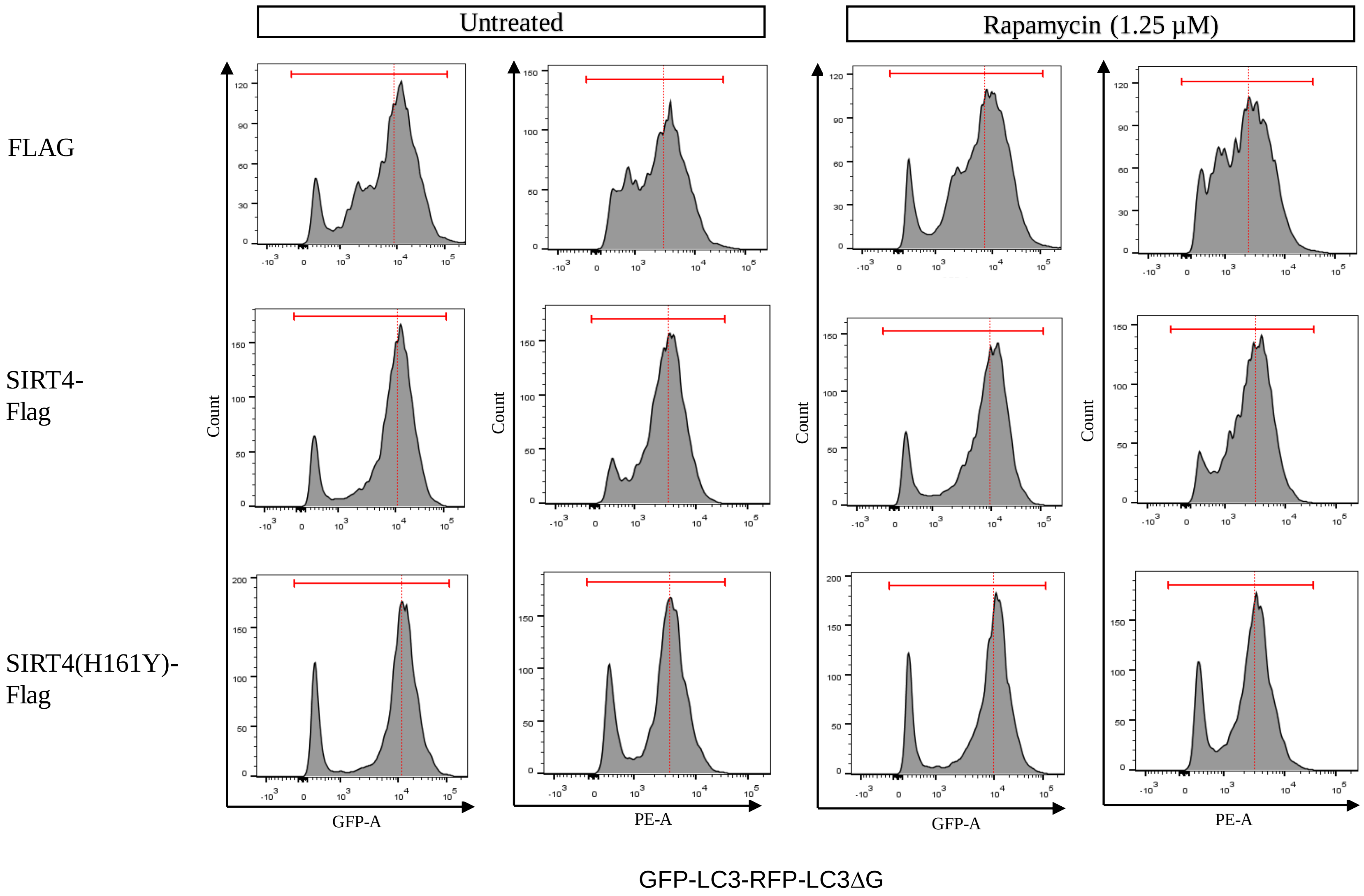

Fig. S9 to S21: Representative data histograms are depicted for all flow cytometry-based experiments to measure autophagic flux (GFP-LC3-RFP-LC3ΔG), mitophagy (mt-mKEIMA), and mitochondrial content (MitoMark). Solid red lines indicate the fluorescence gates chosen for analysis. Red dotted lines represent the median values of emission.

ad Figure S6A

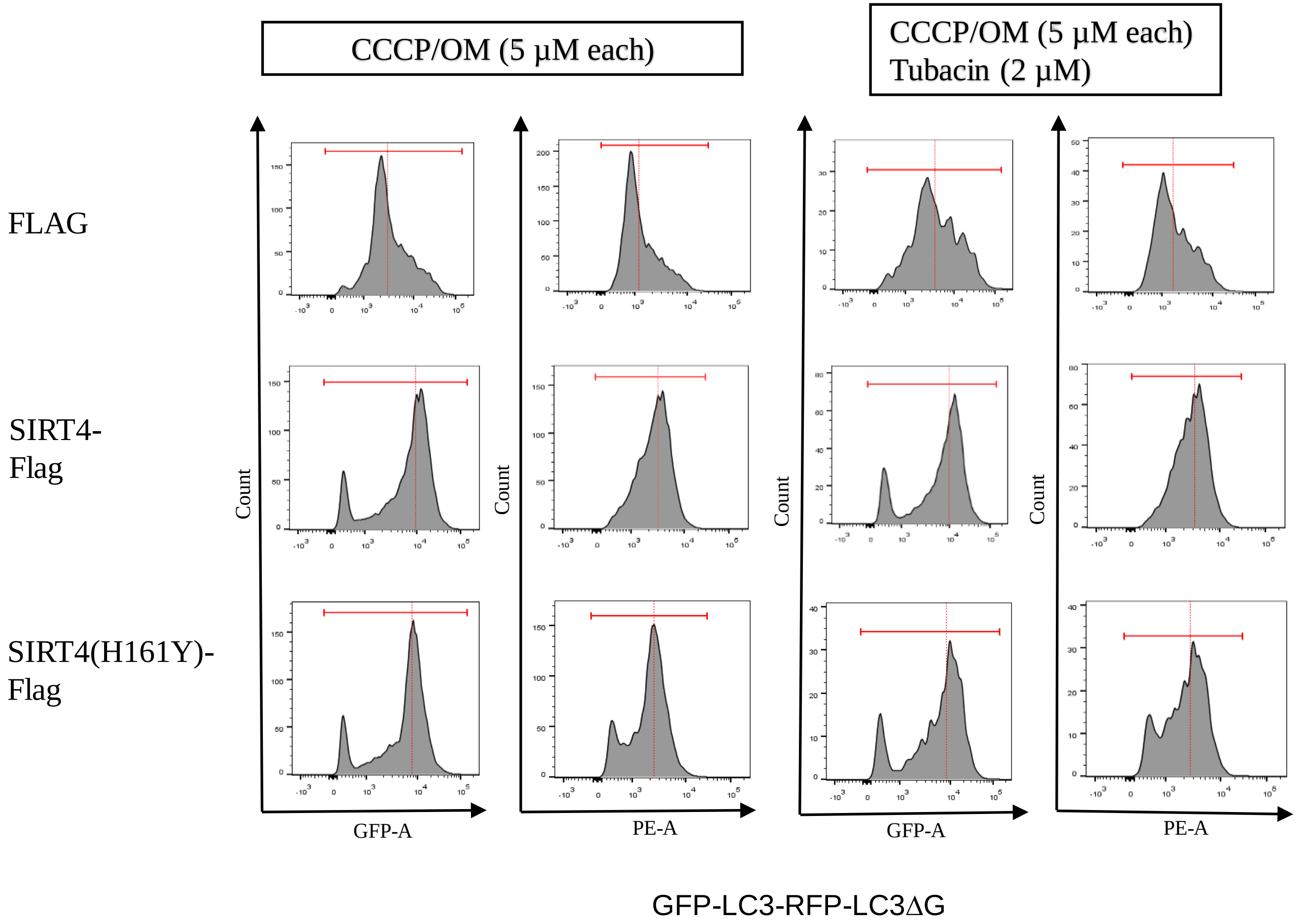

Fig. S9 to S21: Representative data histograms are depicted for all flow cytometry-based experiments to measure autophagic flux (GFP-LC3-RFP-LC3ΔG), mitophagy (mt-mKEIMA), and mitochondrial content (MitoMark). Solid red lines indicate the fluorescence gates chosen for analysis. Red dotted lines represent the median values of emission.

Figure S18

ad Figure S6B

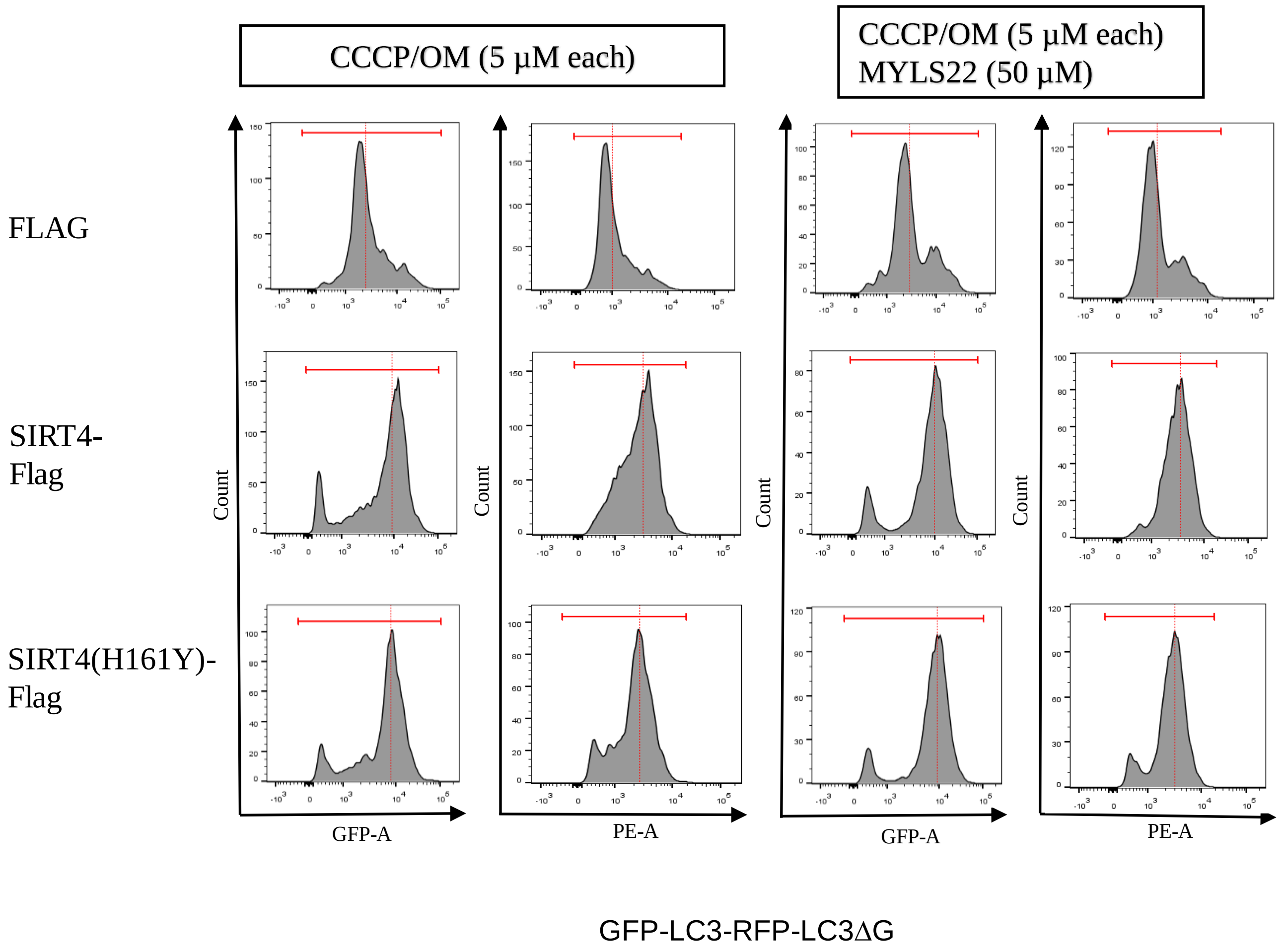

Fig. S9 to S21: Representative data histograms are depicted for all flow cytometry-based experiments to measure autophagic flux (GFP-LC3-RFP-LC3ΔG), mitophagy (mt-mKEIMA), and mitochondrial content (MitoMark). Solid red lines indicate the fluorescence gates chosen for analysis. Red dotted lines represent the median values of emission.

Figure S19

ad Figure S7A

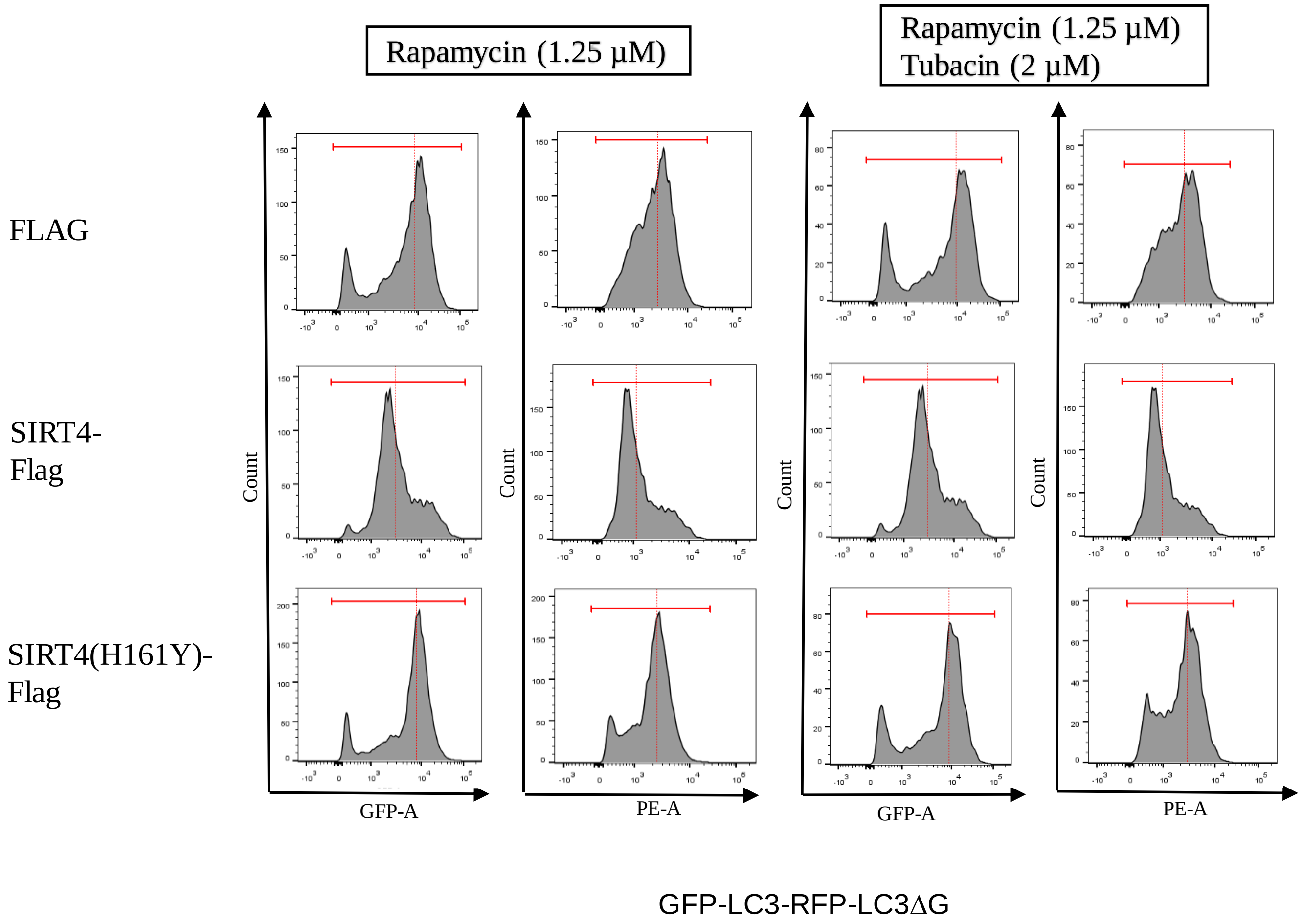

Fig. S9 to S21: Representative data histograms are depicted for all flow cytometry-based experiments to measure autophagic flux (GFP-LC3-RFP-LC3ΔG), mitophagy (mt-mKEIMA), and mitochondrial content (MitoMark). Solid red lines indicate the fluorescence gates chosen for analysis. Red dotted lines represent the median values of emission.

Figure S20

ad Figure S7B

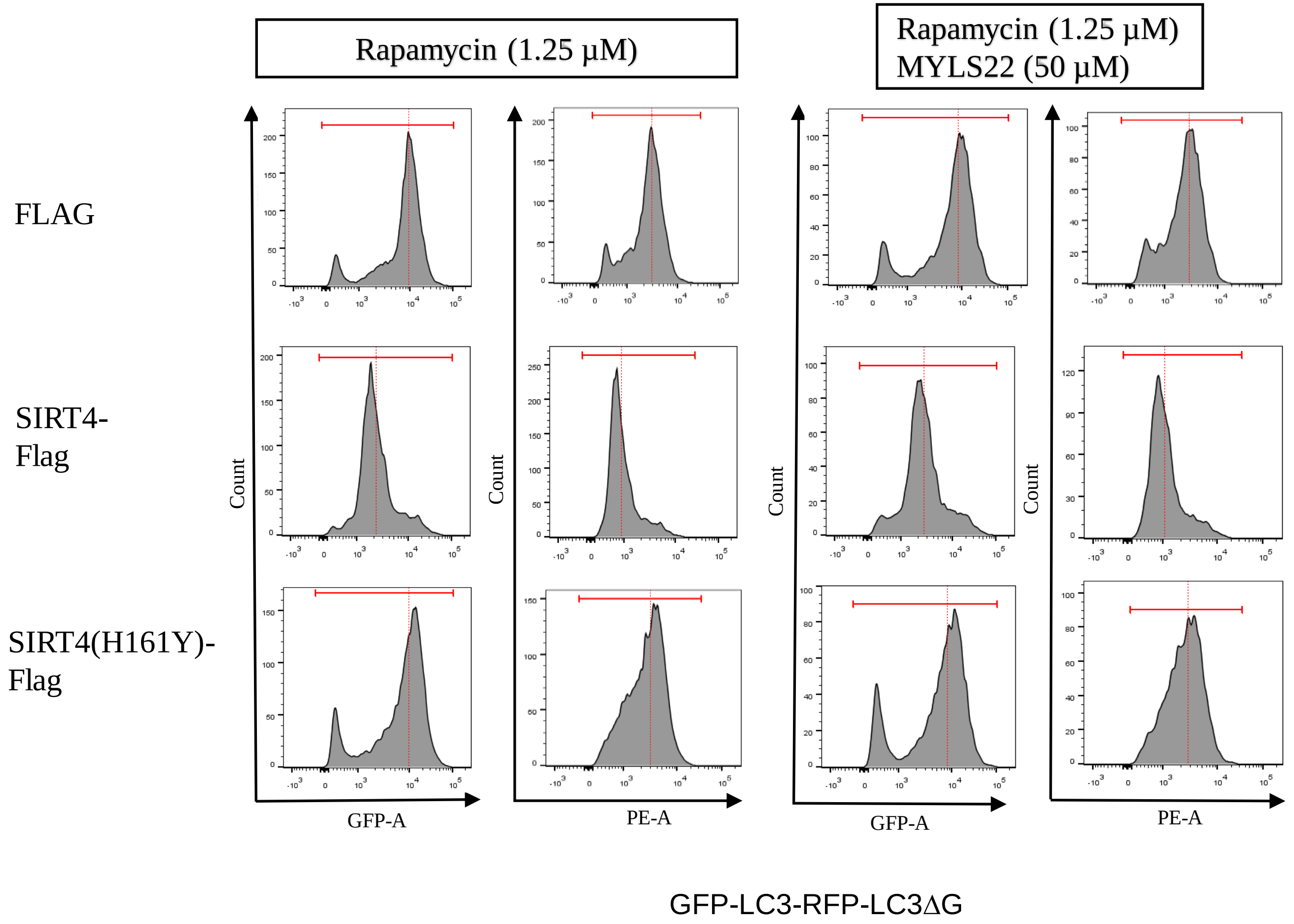

Fig. S9 to S21: Representative data histograms are depicted for all flow cytometry-based experiments to measure autophagic flux (GFP-LC3-RFP-LC3ΔG), mitophagy (mt-mKEIMA), and mitochondrial content (MitoMark). Solid red lines indicate the fluorescence gates chosen for analysis. Red dotted lines represent the median values of emission.

Figure S21
