# Supplementary material for: SIRT4 positively regulates autophagy via ULK1, but independently of HDAC6 and OPA1
Source: FEBS Open Bio. 2025 Nov 20;16(5):870–84. doi: 10.1002/2211-5463.70164 (PMC13145358; doi:10.1002/2211-5463.70164)
Supplement: Supplementary file 2 — Table S1. Primary antibodies used in this study. [file FEB4-16-870-s001.docx]

**Supporting Information**

| **Table S1.** |  |  |  | |  | |
| --- | --- | --- | --- | --- | --- | --- |
| **Primary antibodies** | **Species** | **Dilution** | **Supplier** | **Reference** | |  |
| myc | rabbit | 1:2.000 | Cell Signaling Technology | 2272 | |  |
| eGFP | mouse | 1:2.000 | Roche | 11814460001 | |  |
| eGFP | mouse | 1:20.000 | Proteintech | 66002-1-AP | |  |
| HDAC6 | rabbit | 1:1.000 | Cell Signaling Technology | 7558 | |  |
| acetyl. Tubulin (K40) | mouse | 1:1.000 | Santa Cruz Biotechnology | sc-23950 | |  |
| α-Tubulin | rabbit | 1:1.000 | Abcam | ab52866 | |  |
| α-Tubulin | rabbit | 1:2.000 | Proteintech | 11224-1-AP | |  |
| OPA1 | mouse | 1:1.000 | BD | 612607 | |  |
| HIF1 a | rabbit | 1:1.000 | Abcam | ab179483 | |  |
| LC3 | rabbit | 1:500 | Cell Signaling Technology | 2775 | |  |
| ULK1 | rabbit | 1:1.000 | Cell Signaling Technology | 8054 | |  |
| pS757-ULK | rabbit | 1:1.000 | Cell Signaling Technology | 6888 | |  |
| pS638-ULK | rabbit | 1:1.000 | Cell Signaling Technology | 14205 | |  |
| pS6 (Ser240/244) ribosomal protein | rabbit | 1:1.000 | Cell Signaling Technology | 2215 | |  |
| S6 ribosomal protein | mouse | 1:1.000 | Cell Signaling Technology | 2317 | |  |
| β-Actin | mouse | 1:20.000 | Proteintech | 66009-1-Ig | |  |

Primary antibodies used in this study.
